# Quasiparticle spin resonance and coherence in superconducting aluminium

**DOI:** 10.1038/ncomms9660

**Published:** 2015-10-26

**Authors:** C. H. L. Quay, M. Weideneder, Y. Chiffaudel, C. Strunk, M. Aprili

**Affiliations:** 1 Laboratoire de Physique des Solides (CNRS UMR 8502), Bâtiment 510, Université Paris-Sud 91405 Orsay, France; 2 Institute for Experimental and Applied Physics, University of Regensburg 93040 Regensburg, Germany

## Abstract

Conventional superconductors were long thought to be spin inert; however, there is now increasing interest in both (the manipulation of) the internal spin structure of the ground-state condensate, as well as recently observed long-lived, spin-polarized excitations (quasiparticles). We demonstrate spin resonance in the quasiparticle population of a mesoscopic superconductor (aluminium) using novel on-chip microwave detection techniques. The spin decoherence time obtained (∼100 ps), and its dependence on the sample thickness are consistent with Elliott–Yafet spin–orbit scattering as the main decoherence mechanism. The striking divergence between the spin coherence time and the previously measured spin imbalance relaxation time (∼10 ns) suggests that the latter is limited instead by inelastic processes. This work stakes out new ground for the nascent field of spin-based electronics with superconductors or superconducting spintronics.

## Abstract (web summary)

Conventional superconductors were thought to be spin inert, but long-lived, spin-polarized excitations, or quasiparticles, have recently been observed. Here, the authors demonstrate quasiparticle spin resonance in the mesoscopic superconductor aluminium and estimate the spin coherence time.

## Graphical abstract

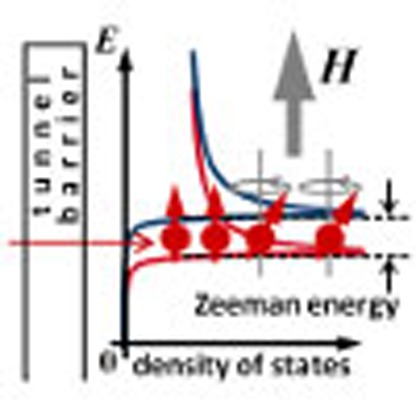

Spin/magnetization relaxation and coherence times, respectively, *T*_1_ and *T*_2_, initially defined in the context of NMR, are general concepts applicable to a wide range of systems, including quantum bits^1^,^2^,^3^,^4^. If one thinks of spins as classical magnetic moments, *T*_1_ is the time over which they align with an external magnetic field, while *T*_2_ is the time over which Larmor-like precessions of the spins around the external field remain phase coherent^2^. (*T*_1_ is sometimes also called the longitudinal or spin-lattice relaxation time and *T*_2_ the transverse relaxation time.) *T*_1_∼*T*_2_ for conduction electrons in most normal metals^3^,^5^,^6^,^7^.

In a typical electron spin resonance (ESR) experiment, electrons are immersed in an external homogenous static magnetic field, *H*. Microwave radiation creates a perturbative transverse magnetic field (perpendicular to the static field) of frequency *f*_RF_. The power *P*(*H*, *f*_RF_) absorbed by the spins from the microwave field is determined, usually by measuring the fraction of the incident microwaves that is not absorbed, that is, either transmitted or reflected. When *H* is tuned to its resonance value, 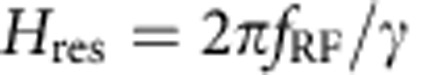
—with *γ* the gyromagnetic ratio—the electron spins precess around *H* and *P*(*H*, *f*_RF_) is maximal. *P*(*H*, *f*_RF_) is proportional to the imaginary part of the transverse magnetic susceptibility, i.e. to 
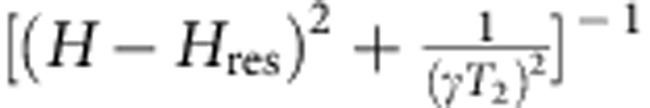
 in the case of a linearly polarized field^8^. Thus, *T*_2_=2/(*γ*Δ*H*), where Δ*H* is the full width at half maximum of the power resonance as a function of *H*.

At first glance, these ideas might seem to be irrelevant to conventional Bardeen–Cooper–Schrieffer (BCS) superconductors, as the BCS superconducting ground state is a condensate of Cooper pairs of electrons with opposite spins (in a singlet state)^9^. It has recently been demonstrated, however, that a non-equilibrium magnetization can appear in the quasiparticle (that is, excitation) population of a conventional superconductor, with *T*_1_ on the order of several nanoseconds^10^,^11^,^12^,^13^,^14^.

This raises the question of *T*_2_ for these non-equilibrium quasiparticles; however, the short penetration depth of magnetic fields in type-I superconductors (∼16 nm for bulk aluminium) creates difficulties for the observation of quasiparticle spin resonance (QSR): firstly, the signal is small—for normal metals, conduction ESR measurements are typically carried out on macroscopic foils tens of microns thick^3^,^15^, and, second, the magnetic field in the superconductor is highly inhomogeneous^16^. (In type-II and unconventional superconductors, the entry of vortices into the sample can solve the first problem but not the second.)

In the following, we overcome these obstacles using thin-film samples and two novel on-chip microwave detection techniques to perform QSR experiments on superconducting aluminium. We find *T*_2_∼100 ps<<*T*_1_∼10 ns (ref. 12), in contrast to normal metals where *T*_1_∼*T*_2_, and identify spin–orbit scattering as the main decoherence mechanism.

## Results

### Devices and measurement set-up

**Figure 1:**
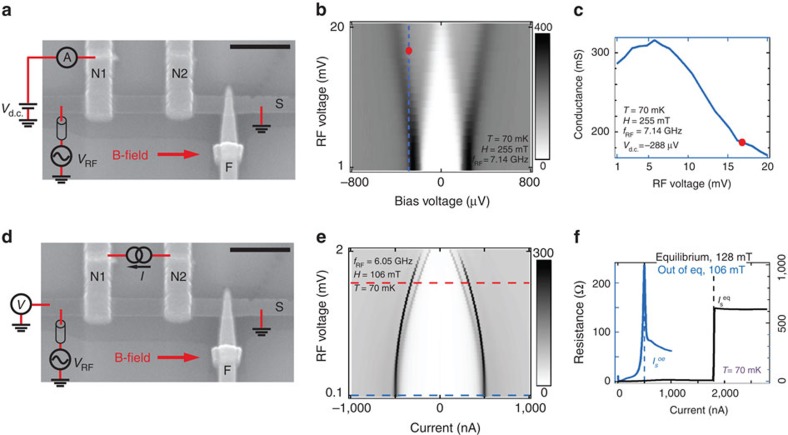
Two on-chip microwave power detection schemes for superconducting (hybrid) devices. (**a**,**c**) Scanning electron micrograph of a device nominally identical to Device A (scale bar, 1 μm) and schematic drawings of the two measurement set-ups. (Data shown are from Device A unless otherwise stated.) In both cases, a static magnetic field, *H* is applied parallel to a superconducting bar (S, Al) and a sinusoidal signal of root mean squared amplitude *V*_RF_ and frequency *f*_RF_ in the microwave range applied across the length of S (with a lossy coaxial cable in series), resulting in a high-frequency field perpendicular to *H*. To detect the spin precession of the quasiparticles in S, two on-chip detection methods are used. (**a**) Detection scheme 1: a voltage *V*_d.c._ is applied between S and a normal electrode (N1, thick Al) with which it is in contact via an insulating tunnel barrier (I, Al_2_O_3_). The differential conductance *G*=d*I*/d*V*_d.c._ is measured, where *I* is the current between N1 and S. (**b**) *G* as a function of *V*_d.c._ and nominal *V*_RF_ (at the output of the generator and not accounting for attenuation in the lines). The red dot indicates the operation point of the detector for the data in Fig. 2: 
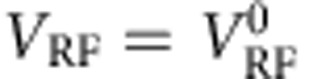
, *V*_d.c._=−288 μV. For any given frequency, we define 
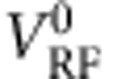
 as the *V*_RF_ (at the output of the generator) at which the effective voltage at the device is the same as that for *f*_RF_=7.14 GHz and *V*_RF_=16.81 mV. (See Supplementary Note 2 and Supplementary Fig. 2). (**c**) A slice of **b** at *V*_d.c._=−288 μV (blue dashed line in **b**) with the operation point indicated. (**d**) Detection scheme 2: a current *I*_d.c._ is injected along the length of S. We measure either the voltage *V* between the ends of the S bar or the differential resistance *R*=d*V*/d*I*_d.c._. We record in particular the switching current *I*_S_ at which S first becomes resistive. (**e**) *R* as a function of *I*_d.c._ and nominal *V*_RF_ (not accounting for attenuation in the lines). The switching current *I*_S_ at which S become resistive appears here as a peak in *R*. *I*_S_ can be seen to decrease monotonically with *V*_RF_. The red dashed line indicates the operation point of the detector for the data in Fig. 3: *V*_RF_=0.8
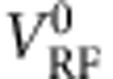
. (**f**) The blue trace is the first slice of **e** (blue dashed line in **e**) at *V*_RF_=0.1 mV. The black trace is a two terminal measurement of the S bar, in the absence of microwaves, with a constant corresponding to the resistance of the lines subtracted. The difference in *I*_S_ between the two indicates that the S bar is strongly out of equilibrium in our second (switching current) detection scheme. (See Supplementary Note 4).

Our devices are thin-film superconducting (S) bars, with a native insulating (I) oxide layer, across which lie normal metal (N) and either superconducting (S′) or ferromagnetic (F) electrodes (in the cases of devices B and A, respectively). Here S and S′ are both aluminium, I is Al_2_O_3_, F is cobalt with an aluminium capping layer and N is thick aluminium with a critical magnetic field of ∼50 mT (ref. 17). In all the data shown here, the N electrodes are in the normal state. The F electrodes are not used here, but rather in frequency-domain measurements of *T*_1_ reported elsewhere^10^. Device A, lying atop a Si/SiO_2_ substrate, is shown in Fig. 1. As in previous experiments, the NIS junctions have ‘area resistances' of ∼6 × 10^−6^ Ω cm^2^ (corresponding to barrier transparencies of ∼1 × 10^−5^) and tunnelling is the main transport mechanism across the insulator. (See Supplementary Information of ref. 12.) Measurements were performed at temperatures down to 60 mK, in a dilution refrigerator. On the basis of conductance measurements across the NIS juctions, S has a superconducting gap of 205±10 μV (265±10 μV) in device A (device B), corresponding to critical temperatures of 1.34±0.07 K (1.75±0.07 K) in the BCS theory.

A static magnetic field *H* is applied in the plane of the device and parallel to the S bar (Fig. 1a). S has a thickness of *d*∼8.5 nm (6 nm) for device A (device B), well within the magnetic field penetration depth *λ*, which we expect to be 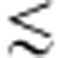
315 nm (375 nm) in our samples at 70 mK. (See Supplementary Note 1 and ref. 18 for details on this estimate.) The ratio of the orbital energy 
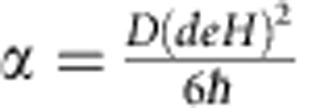
 to the Zeeman energy 
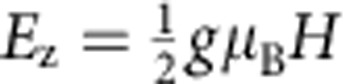
 is ∼0.32 (0.22) for the quasiparticles in S in device A (device B) at 0.5 T 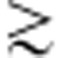 the highest measured resonant magnetic field *H*_res_. It is lower at lower fields. Therefore, the Zeeman energy is always dominant and we are in the ‘paramagnetic limit'^16^,^19^,^20^. Here *D* is the diffusion constant, *e* the electron charge, *g* the Landé *g*-factor, *μ*_B_ the Bohr magneton and *ℏ* Planck's constant. (See Supplementary Note 1 for details.) The data shown below are from device A unless otherwise stated.

A sinusoidal radio frequency signal of frequency 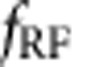 and root mean squared amplitude *V*_RF_ is applied across the length of the S bar (via a lossy, that is, resistive coaxial cable). The resulting supercurrent flowing along the length of S serves primarily to produce the desired high-frequency magnetic field perpendicular to *H*; secondarily, it also breaks some Cooper pairs and thus increases the quasiparticle population. Microwave radiation due to the supercurrent thus impinges on the quasiparticle spins in S. Some of this radiation is absorbed by the quasiparticle spins, and the rest transmitted to and absorbed by the surrounding environment. The ‘transmitted radiation' can appear as a voltage across a tunnel junction between S and N; this is the basis of our first detection scheme (DS1; Fig. 1a). It can also be absorbed by the superconducting condensate, thus reducing the density of Cooper pairs and the current *I*_S_ at which S becomes resistive, known as the switching current; this is the basis of our second detection scheme (DS2; Fig. 1c). Both of our detection schemes for *P*(*H*, *f*_RF_) are therefore entirely ‘on-chip'.

In DS1 (Fig. 1a), we apply a bias voltage *V*_d.c._ across an NIS junction and measure its differential conductance *G*=d*I*/d*V*_d.c._ using standard lock-in techniques. (*I* is the current across the junction.) Figure 1b shows such traces as a function of *V*_RF_, in which we see a flattening of the coherence peaks in a monotonic manner. (This is similar to the effect of classical rectification^10^.) Figure 1c shows a slice of Fig. 1b at *V*_d.c._=−288 μV. *G* across the junction can be seen to be an effective microwave power meter at the chosen operating point (red dot). We define 
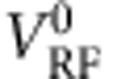
 (for any given frequency) as the reference *V*_RF_ (at the output of the generator) at which the effective voltage at the device is the same as that for *f*_RF_=7.14 GHz and *V*_RF_=16.81 mV. (See Supplementary Note 2 and Supplementary Fig. 2).

In DS2 (Fig. 1d), we measure the voltage–current characteristic of the S bar and record the switching current *I*_S_. A current *I*_d.c._ is injected from one N electrode to another and the resulting voltage *V* across the length of the bar is measured. Figure 1e shows the differential resistance *R*=d*V*/d*I*_d.c._ of the S bar as a function of *I*_d.c._ and of *V*_RF_. The peaks in these traces correspond to *I*_S_. *I*_S_ can be seen to depend monotonically on *V*_RF_ and is thus also a good measure of the latter.

Our on-chip detection provides improved sensitivity compared with earlier work on the spin resonance of conduction electrons in normal metals (CESR)^3^,^21^. Indeed, based on calculations for CESR measurements on macroscopic samples, it was previously thought that CESR signals in type-I superconductors would be unmeasurably small^22^. This is no doubt why, while a considerable amount of work has been done on the CESR in normal metals since the 1950s, to our knowledge only one such measurement has been performed on a bulk BCS superconductor (Nb) in the vortex state, close to the critical field^23^,^24^,^25^.

### Quasiparticle spin resonance

Having characterized our two microwave power meters, we now perform QSR measurements using each of them in turn, and compare the results of both.

**Figure 2:**
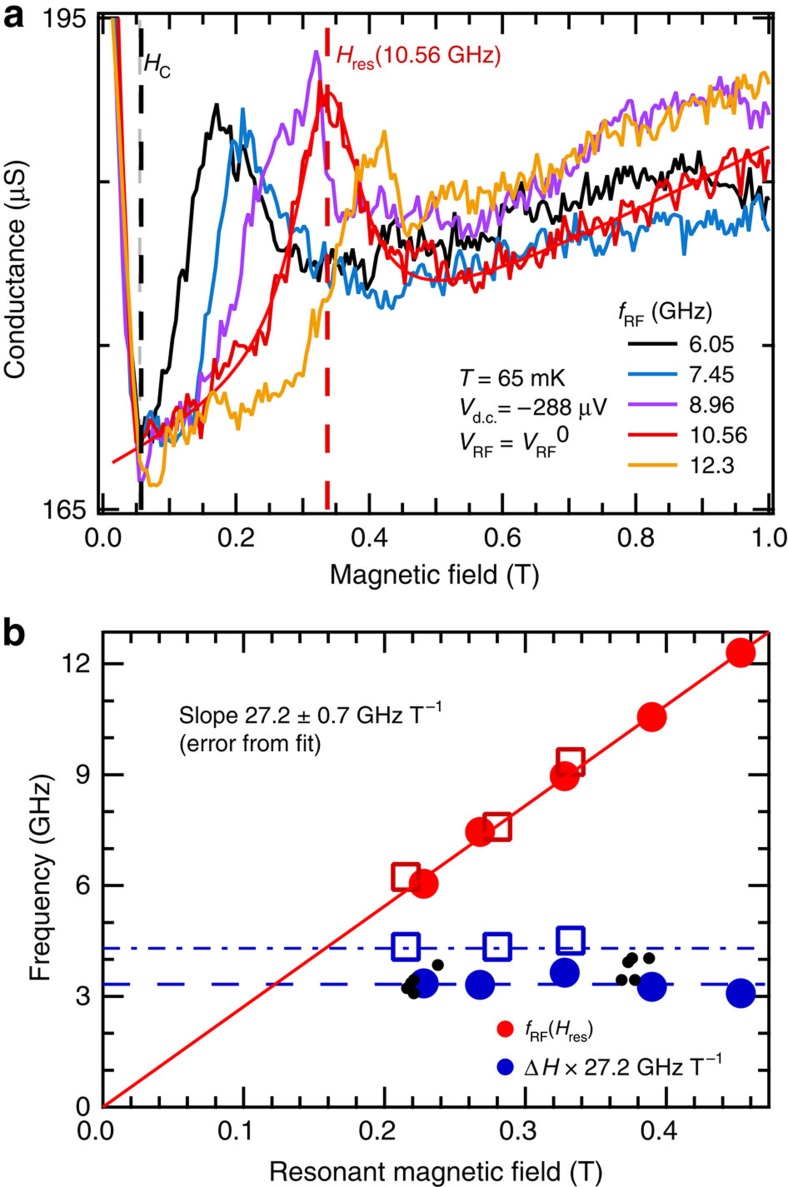
Spin resonance in conductance across tunnel junction. (**a**) NIS junction conductance *G* as a function of *H* at *V*_d.c._=−288 μV and 
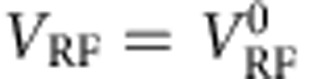
 for different *f*_RF_. The black vertical line indicates the critical field of N. *H*_res_ and Δ*H* are obtained for each *f*_RF_ by fitting a Lorentzian with a linear background. The fit for *f*_RF_=10.56 GHz is shown (thin red line) and *H*_res_ indicated with a red vertical line. (**b**) *H*_res_ and Δ*H* the resonance linewidth (full width at half maximum) as a function of *f*_RF_ (red and blue circles, respectively). A linear fit to *H*_res_(*f*_RF_) data gives a Landé *g*-factor of 1.95±0.2. The black dots indicate values obtained at different powers or with the second detection scheme. (See Supplementary Note 3 and Supplementary Fig. 3). All dots and circles have been offset by 53 mT to account for a systematic shift in the applied magnetic field during the associated cooldown. The squares indicate values obtained from Device B, in which S is 6-nm thick.

In the first set of measurements, using DS1, we operate the NIS junction detector—which is to say measure the differential conductance *G*=d*I*/d*V*_d.c._ across it—at a fixed *V*_d.c._ of −288 μV and 
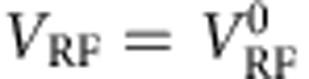
. (*G*(*V*_d.c._, *H*) traces in the absence of microwaves are shown in Supplementary Fig. 1). As can be seen in Fig. 1c, at this operation point, small decreases in the absorbed power will result in a proportional increase in *G*. (It can also be seen that we remain in the linear regime in the measurements in Fig. 2).

Figure 2a shows *G*(*H*) at several different *f*_RF_. As expected, each trace shows a resonance, that is, an increase in *G*(*H*) due to the fact that more power is being absorbed by precessing quasiparticle spins and therefore less appearing across the NIS junction. We determine *H*_res_ and Δ*H* by a Lorentzian with a linear-in-*H* background signal to these data. The background comes from magnetic-field-induced orbital depairing in the quasiparticle density of states. This measurement was repeated at different *f*_RF_; *H*_res_ as a function of *f*_RF_ is shown in Fig. 2b. A linear fit to the data gives a *g*-factor of 1.95±0.2, consistent with previous measurements of electrons in Al in the normal state^26^,^27^.

Note that Δ*H* may be larger than its intrinsic value if, for instance, the static field is inhomogeneous in the region of interest^2^. This would then lead to an underestimate of *T*_2_. In our samples, however, *d*/2<<*λ*, as mentioned above. Thus, the magnetic field seen by the quasiparticles is homogeneous to 
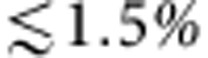
 in the superconductor, much smaller than the Δ*H* we measure, and our estimate of *T*_2_ is unaffected by magnetic field inhomogeneity. This is confirmed by the fact that Δ*H* does not depend on *H*_res_, as can be seen in Fig. 2b. Field homogeneity has been a challenge for both ESR and NMR measurements performed on macroscopic type-II superconductors. In these, specimen dimensions greater than *λ* mean that the field decays significantly within the specimen, and additional complications often arise from the presence of vortices.

**Figure 3:**
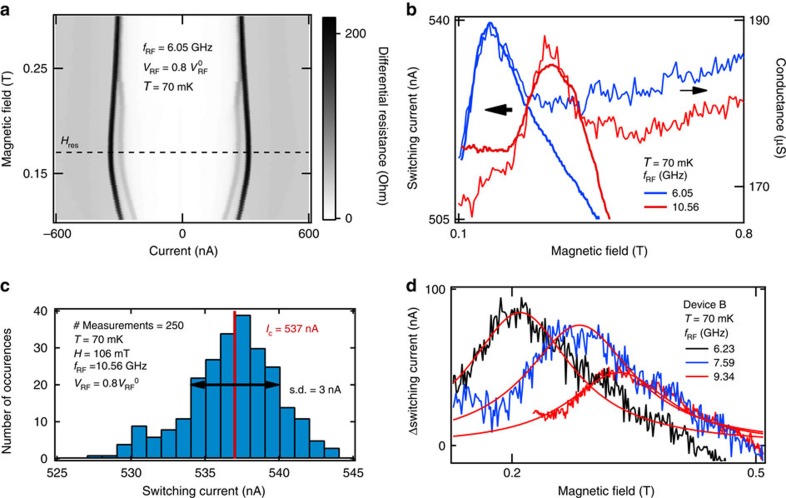
Spin resonance in supercurrent, comparison of detection schemes. (**a**) Differential resistance *R* of the S bar as a function of *H* and *I*_d.c._ with *f*_RF_=6.05 GHz, *V*_RF_=0.8 
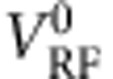
. At *H*_res_∼0.17 T, the resonant field, the switching current *I*_S_ can be seen to increase, indicating that less microwave power is being transmitted to the superconducting condensate as more power is absorbed by the quasiparticles in S. (**b**) Switching current *I*_S_ as a function of static magnetic field *H* for two different *f*_RF_. (Here a slight change was made to the measurement circuit: With reference to Fig. 1d, the current is applied between N2 and F instead of between N1 and N2, hence the slightly higher *I*_S_: the current injection electrodes are closer together.) Superimposed on these traces are the conductance traces from Fig. 2a at the same fields. *H*_res_ and Δ*H* can be seen to be similar for both measurement methods. The bold red trace has been offset downwards by 19 nA. (**c**) The switching currents in **b** are averages of ∼200 measurements, with a s.d. of ∼3 nA. Here we show a histogram of 250 switching currents corresponding to the first point in the bold red trace in **b**. Current was driven long the length of the S bar and the voltage measured between N1 and N2. (Voltage and current leads are thus switched with respect to **b**). (**d**) Device B: switching current *I*_S_ as a function of static magnetic field *H* for three different *f*_RF_, with a linear background subtracted (thick lines). *H*_res_ and Δ*H* obtained from the fits (thin red lines) are shown in Fig. 2b. These *I*_S_ values are averages of ∼500 measurements.

In the second set of measurements, we use DS2. As can be seen in Fig. 1e, small decreases in the absorbed microwave power will result in a proportional increase in the switching current *I*_S_. We first measure the differential resistance *R*=d*V*/d*I*_d.c._ of the S bar as a function of *I*_d.c._ and of *H* at *f*_RF_=6.05 GHz, *V*_RF_=0.8 
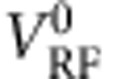
 (Fig. 3a). We observe an increase in *I*_S_ at *H*=0.17 T, which we identify as *H*_res_—at this field, the quasiparticle spins enter into resonant precession, thus absorbing more microwave power. Less power is then transferred to the superconducting condensate and *I*_S_ increases.

In Fig. 3b, we show *I*_S_ as a function of *H* at two different frequencies. (*I*_S_ is the average of 200 switching current values obtained from *V*(*I*_d.c._) measurements.) The expected resonance appears at both frequencies. To compare results from the two different detection schemes, we superimpose on these traces data from Fig. 2a at the same *f*_RF_. We see that both *H*_res_ and Δ*H* are the same for both detection schemes. We also verified that *H*_res_ and Δ*H* are independent of *V*_RF_ (see Supplementary Note 3, Supplementary Fig. 3 and black dots in Fig. 2b).

As DS2 is sensitive to a longer portion of the S bar compared with DS1, the agreement between the two detection schemes is further confirmation that the magnetic field is quite homogenous along the entire length of the S bar between the two N electrodes. Thus, our results for *T*_2_ reported below should be reasonably close to the intrinsic value.

The spin coherence time 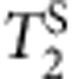 for 8.5-nm-thick superconducting aluminium (device A) is 95±20 ps as determined from Δ*H* (the full width at half maximum of the resonance) in Figs 2a and 3b; this is fairly constant within the range of accessible fields (Fig. 2b). Measurements on device B, in which S is 6-nm thick yielded 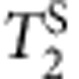
=70±15 ps (Figs 2b and 3d). Both the order of magnitude of 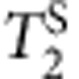, as well as the fact that it is inversely proportional to the film thickness, are consistent with spin coherence limited primarily by 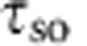, the Elliott–Yafet spin–orbit scattering time^5^,^12^,^26^,^27^,^28^,^29^,^30^,^31^,^32^,^33^,^34^,^35^,^36^.


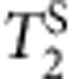
 is, however, relatively unaffected by the quasiparticle density: In device A, the linewidths measured by DS1 and DS2 are identical (Fig. 3b), whereas the quasiparticle density is estimated to be about two orders of magnitude higher in the supercurrent measurements (with injection across the tunnel junctions) than in the conductance measurements. (See Fig. 1f, Supplementary Note 4 and ref. 37.) In device B, the linewidth remained unchanged at temperatures of up to 600 mK and at injection currents (across an NIS junction) of up to 42 nA.

### Equilibrium measurement of the spin-flip time

**Figure 4:**
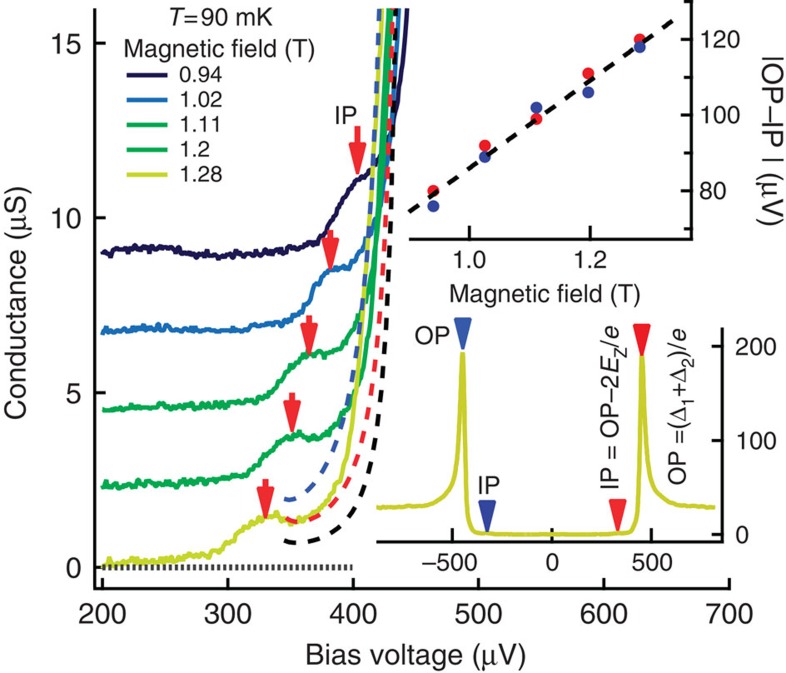
Independent measurement of spin mixing due to the spin–orbit interaction. Device B: conductance *G* as a function of voltage *V*_d.c._ across an SIS′ junction, at different magnetic fields *H* (offset by 2.2 μS). Apart from the principal, outer peak (OP) at *V*_d.c._=(Δ+Δ′)/*e*, with Δ (Δ′) the superconducting energy gap of S (S′), a smaller, inner peak (IP) can be seen at ∼*V*_d.c._=(Δ+Δ′−2*E*_Z_)/*e*, with *E*_Z_ the Zeeman energy. Fitting the data at *H*=1.28 T to numerical calculations based on refs 20, 38 (red dotted line) yields a spin–orbit time 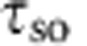 of 45±5 ps. Numerical results for 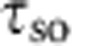
=23 and 69 ps are also shown (blue and black dotted lines, respectively). Lower inset: full-conductance trace at *H*=1.28 T, showing all peaks. Upper inset: distance in *V*_d.c._ between outer and inner peaks at positive (red dots) and negative (blue dots) energies. The black dotted line, which has a slope of 2*E*_Z_/*e*, is a guide to the eye.

It is thus reasonable to compare 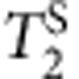 with an independent estimate of 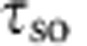 from *G*(*V*_d.c._) measurements across an SIS′ junction in device B, following work by Tedrow and Meservey on Al thin films^20^,^31^,^38^. (Here S′ is a 8.5-nm-thick superconducting Al counter electrode.) In the absence of spin–orbit coupling, as spin is conserved in tunnelling between S and S′, *G*(*V*_d.c._) shows a peak at *V*_d.c._=(Δ+Δ′)/*e* at all fields (with Δ (Δ′) the superconducting energy gap of S (S′)) and the Zeeman effect is effectively invisible. In the presence of small but finite spin–orbit coupling, spin mixing modifies the density states for each spin and leads to a small conductance peak at a lower voltage *V*_d.c._=(Δ+Δ′−2*E*_z_)/*e*, whose height ∼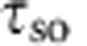 (Fig. 4). (At very strong spin–orbit coupling, the two peaks merge; spin orbit then has the same effect on the conductance trace as a depairing magnetic field^20^.) A fit using the BCS density of states and the theory outlined in refs 20, 38 yields a spin-mixing parameter *b* of 0.018±0.002 and 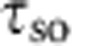
=45±5 ps, which agrees well with 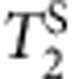 above, as well as previous measurements^31^ (Fig. 4).

## Discussion

We now address the conspicuous divergence between 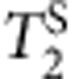 and 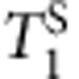, the latter previously determined to be on the order of ∼10 ns in films of similar thickness^12^. In comparison, in normal Al, 
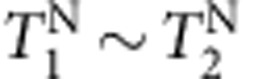
∼50 ps in such films at 4 K (ref. 12). (Note that 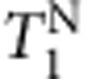 depends on the film thickness^12^,^26^,^27^,^33^,^34^,^35^,^36^).

The striking effect can be understood by considering that spin imbalance (that is to say non-equilibrium magnetization) in superconductors can be thought of, in a simple picture, as having contributions from both a spin-dependent shift in the quasiparticle chemical potentials as well as spin-independent heating of the quasiparticle population^11^,^39^,^40^. The former can be characterized by 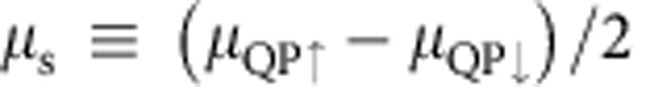
—with *μ*_QPα_ the chemical potential of the spin-α quasiparticles—and the latter by an effective temperature T*. (A more general description could be based on, for example, the quasiparticle distribution function for each spin).

T* and *μ*_s_ should relax on different timescales, respectively, the inelastic scattering time 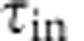 and the spin-mixing time 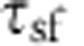 (dominated in thin-film Al by 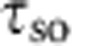). 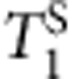 is then the longer of 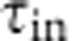 and 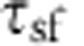, whereas 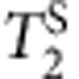 should be affected only by 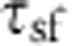 but not 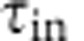
–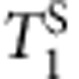 and 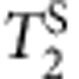 in superconductors can thus be quite different. That the previously determined value for 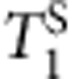 is close to the inelastic relaxation time in Al, estimated to be ∼5 ns from ref. 41 is consistent with spin relaxation being limited by 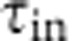 and in particular quasiparticle–quasiparticle interactions^41^,^42^. We note that the quasiparticle–phonon interaction time 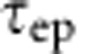
—over which the whole system relaxes to equilibrium—has recently been determined in frequency-domain measurements in 20-nm-thick Al films at 100 mK to be ∼1.6 μs (ref. 43), much longer than both 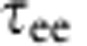 and 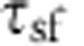. Thus, 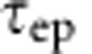 should not be a limiting timescale for either 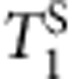 or 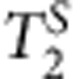.

In contrast, spin accumulation in normal metals is impervious to T* and is due only to *μ*_s_. Spin relaxation in normal Al then occurs over 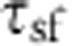, which also governs spin coherence. Thus, 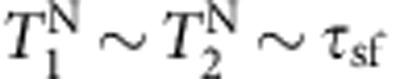 and indeed these terms are sometimes used interchangeably in the literature.

Our results are, in sum, consistent with spin–orbit scattering as the main spin decoherence mechanism in thin-film superconducting Al, and also with a picture in which *T*_1_ and *T*_2_ diverge in superconductors due to the plural sources of spin accumulation in these systems. This has implications for (coherent) computing possibilities using superconducting spintronic devices^44^, and also raises new questions about the interactions between the two sources of spin imbalance in the simple picture presented above, as well as of both with the superconducting condensate^39^,^40^.

Our methods for measuring the coherence time can in principle be extended to other superconducting materials—both conventional and unconventional—as long as they can be nanostructured. A little more speculatively, our work also calls to mind the NMR experiments performed on superfluid ^3^He, which were critical for identifying its different phases and in particular their spin (triplet) structure; it opens up analogous perspectives in (unconventional) superconductivity, where (the manipulation of) the internal structure of Cooper pairs is now an active field of enquiry.

## Methods

### Sample fabrication and transport measurements

We fabricate our samples with standard electron-beam lithography and angle evaporation techniques in an electron-beam evaporator with a base pressure of 5 × 10^−9^ mbar. We first evaporated ∼6 nm (∼8.5 nm) of Al for device B (A), which is then oxidized at 8 × 10^−2^ mbar for 10′ to produce a tunnel barrier, then 100 nm of Al at an angle. For device B, we then evaporated 8.5 nm at another angle. (In device A, 40 nm of Co and 4.5 nm of Al are evaporated at the second angle, but these electrodes were not used in this work.) All transport measurements were carried out in a ^3^He–^4^He dilution refrigerator with a base temperature of 60 mK. Differential resistances were measured with standard lock-in techniques. The switching currents *I*_S_ reported here are the mean values of 200–500 measurements.

## Additional information

**How to cite this article:** Quay, C. H. L. *et al.* Quasiparticle spin resonance and coherence in superconducting Aluminium. *Nat. Commun.* 6:8660 doi: 10.1038/ncomms9660 (2015).

## Supplementary Material

Supplementary Information Supplementary Figures 1-4, Supplementary Notes 1-4 and Supplementary References
