# Supplementary material for: Quasiparticle spin resonance and coherence in superconducting aluminium
Source: Nat Commun. 2015 Oct 26;6:8660. doi: 10.1038/ncomms9660 (PMC4639902; doi:10.1038/ncomms9660)
Supplement: Supplementary Information — Supplementary Figures 1-4, Supplementary Notes 1-4 and Supplementary References [file ncomms9660-s1.pdf]

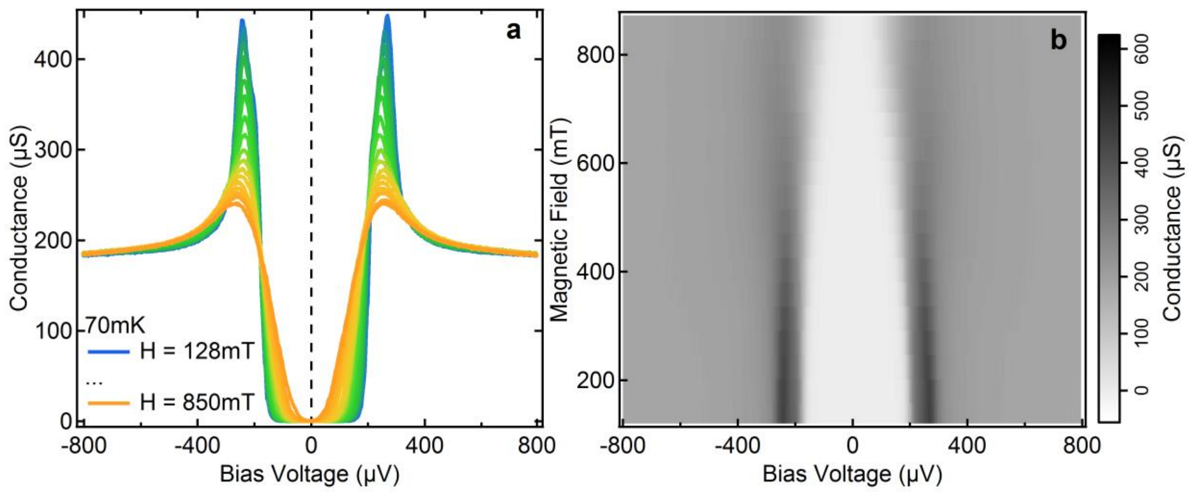

Supplementary Figure 1| Conductance-Voltage Traces in the Absence of Microwaves. Conductance of the junction N1-S, used as the detector for Figure 2 of the main text, as a function of bias voltage and magnetic field, above the critical field of N1 and in the absence of microwaves. (N1 is normal for all the data shown in and relevant to the points made in the main manuscript.) The same data are plotted in two different ways. The conductance spectra can be seen to vary smoothly as a function of magnetic field.

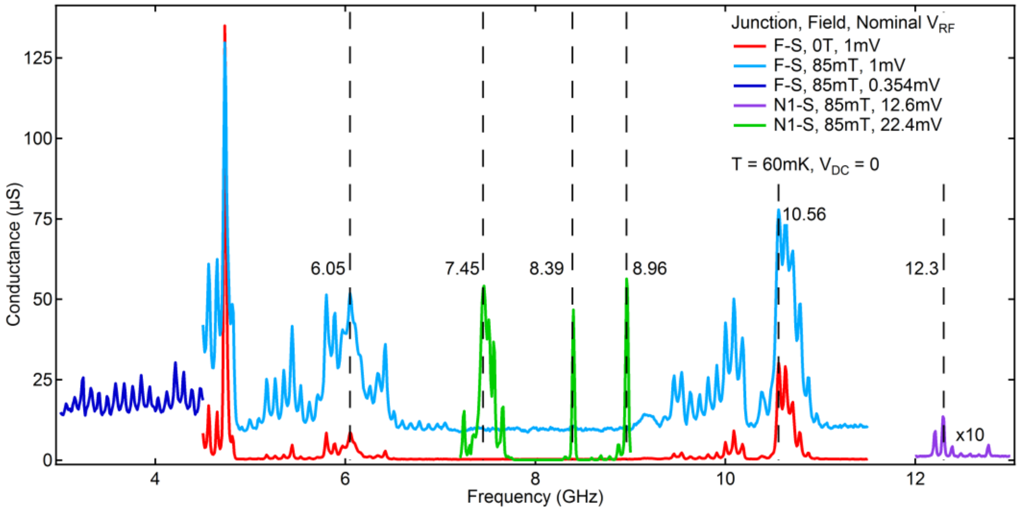

Supplementary Figure 2| Zero-bias conductance of two different junctions as a function of frequency for several different nominal microwave voltages (at the output of the generator).

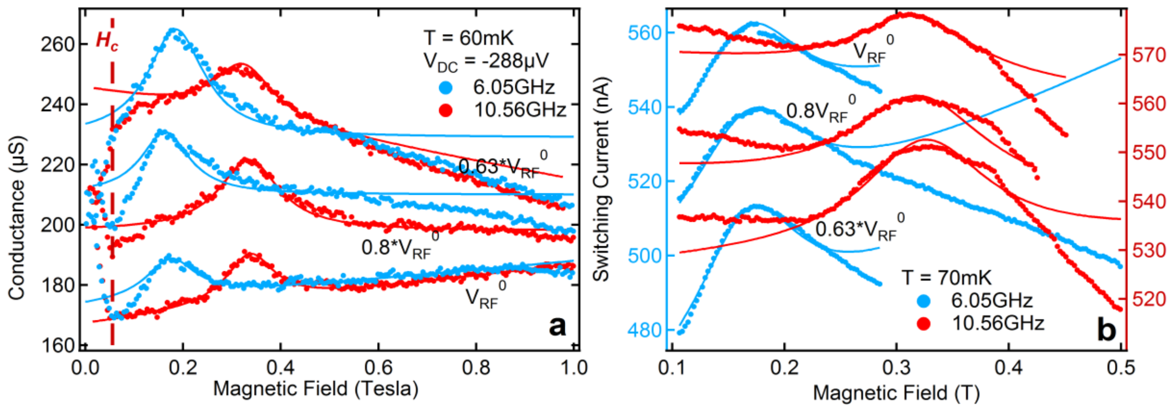

Supplementary Figure 3| Quasiparticle spin resonances at different microwave voltages, measured with. (**a**) Detection Scheme 1. (**b**) Detection Scheme 2. For DS2, the measurement circuit is the same as in Figure 3b of the main text. Solid lines are fits of Lorentzians with a linear background.

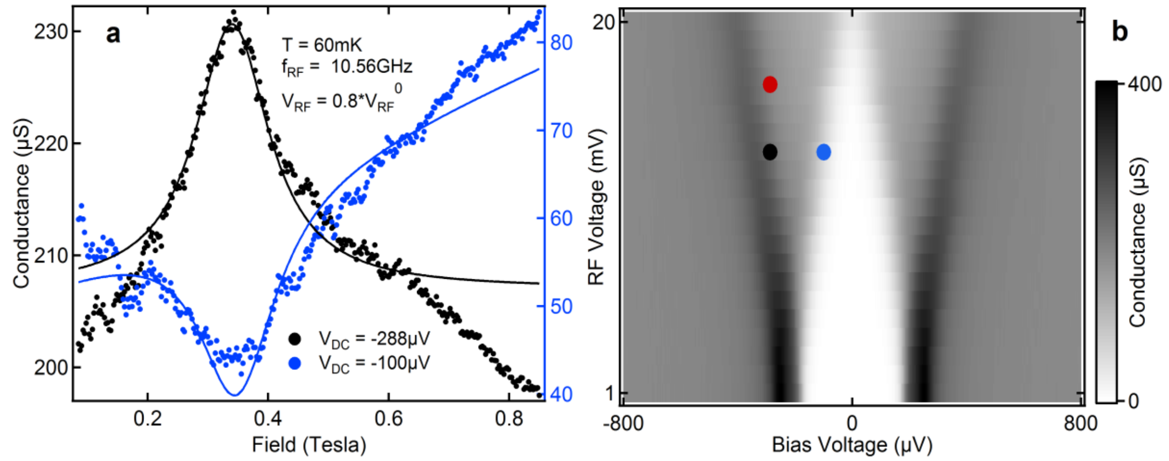

Supplementary Figure 4| (**a**) The quasiparticle spin resonance measured with Detection Scheme 1 at the operating points shown in (b). (**b**) The colour-coded operating points for the blue and black traces shown in (a). The red dot is the operating point for the data shown in Figure 2a of the main text.

Supplementary Note 1: Estimates of D, λ and α in the superconducting Al films

The conductivity σ of a diffusive metal is related to the diffusion constant D by $\sigma = e^2ND$, where e is the charge of the electron and N the density of states [1]. We obtain $\sigma = 8.2 \times 10^6$S·m^{-1} for Device A from the blue trace in Figure 1f of the main text, taking the relevant volume to be that of the S bar between the electrodes N1 and N2 from centre to centre. Using $N = 2.4 \times 10^{22}$ states per eV per cm^3 for aluminium, we obtain $D = 2 \times 10^{-3}$m^2·s^{-1}.

The penetration depth of the magnetic field into the superconductor is $\lambda = \sqrt{\frac{\hbar}{\mu_0 \pi \sigma \Delta}} \sim$ 315nm for Device A [2]. Here μ_0 is the vacuum permittivity, $\hbar$ Planck's constant and Δ the zero-temperature superconducting gap. λ is much greater than the thickness of our Al film, d~8.5nm. If we assume that the mean free path is limited by d, then in the Drude model the conductivity should simply scale as $1/d$ and Device B, in which d~6nm, should have $\lambda \sim$375nm.

The orbital energy of electrons in diffusive thin films with a magnetic field H applied in the plane of the film is $\alpha = \frac{D(deH)^2}{6\hbar}$ whereas the Zeeman energy is $E_z = \frac{1}{2} g \mu_B H$, with g the Landé *g*-factor and μ_B the Bohr magneton [3, 4]. (Note that Ref. [3] uses cgs units and Ref. [4] is missing a factor of $\hbar$. The expressions given here are correct and in SI units.) At the highest resonant field (δ0.5T) measured in this work, we have α/E_z~0.32 for Device A and ~0.22 for Device B. We are thus always in the 'paramagnetic limit', where the Zeeman effect dominates over orbital depairing.

Supplementary Note 2: Choice of Frequencies

As in our previous work [5], Figures 1b and 1e of the main text are reproducible at any frequency modulo a constant shift in the $\boldsymbol{V_{RF}}$ axis, with $\boldsymbol{V_{RF}}$ being nominal microwave voltage (i.e. the voltage at the output of the generator). This constant shift is due to the frequency-dependent attenuation of our microwave line (greater attenuation at higher frequencies) as well as resonances in the line. The conductance of a junction at zero bias is thus a measure of the microwave power arriving at the junction/device.

As noted in our main text, we define V_{RF}^0 (for any given frequency) as the nominal V_{RF} at which the effective voltage at the device is the same as that for f_{RF} = 7.14GHz and V_{RF} = 16.81mV.

To select the frequencies at to search for the quasiparticle spin resonance we measure the conductance of a junction as a function of frequency at various V_{RF} and at $V_{DC} = 0$. (Figure 2) As can be seen in Figure 1b of the main text, the conductance at $V_{DC} = 0$ has a monotonic dependence on the effective V_{RF} at the device and can be taken as an indication of the latter. The effect of the frequency-dependent transmission of our microwave line is quite apparent in these data.

For the measurements shown in the main text, we selected f_{RF} at which the conductance is at a locally maximal, corresponding also to local maxima in the real microwave voltage at the device as a function of f_{RF}. We do this to avoid any experimental missteps accidentally delivering more power to the device than required, thus possibly blowing it up. In addition, this avoids

unnecessary dissipation of energy in the microwave line, which if excessive could lead to a rise in the base temperature of the dilution refrigerator. (We did not notice this in our measurements.)

Supplementary Note 3: Quasiparticle Spin Resonance, Dependence on Microwave Amplitude (Both Detection Schemes) and on (Detection Scheme 1)

As explicated in the main text, the 'operating point' of Detection Scheme 1 (DS1) is defined by the chosen values of V_{RF} and V_{DC} whereas the operating point of Detection Scheme 2 (DS2) is defined by the chosen value of V_{RF}. We show here that our results for resonant field H_{res} and the resonance linewidth ΔH are robust against the choice of operating point in both detection schemes.

We first show that our results from both detections schemes are independent of V_{RF}. Supplementary Figure 3 shows the resonances from Figure 3b of the main text, together with the same measurements taken at different V_{RF}. Traces taken with DS1 are shown in Supplementary Figure 3a while those taken with DS2 are shown in Supplementary Figure 3a. Visually, H_{res} and ΔH are not significantly affected by the microwave voltage. The values for H_{res} and ΔH that we obtain from the fits shown here are plotted in Figure 3c of the main text (black dots).

Next, we show that our results from DS1 are independent of the choice of V_{DC}. In Supplementary Figure 4a, we show a measurement of conductance as a function of applied magnetic field at $V_{\mathrm{RF}} = 0.8V_{\mathrm{RF}}^{0}$, V_{DC}= -288μV (black trace in 4a, black dot in 4b) together with the same measurement taken at $V_{\mathrm{RF}} = 0.8V_{\mathrm{RF}}^{0}$, V_{DC}= -100μV (blue trace in 4a, blue dot in 4b). For the black trace we have H_{res} = 340mT±5mT and ΔH = 148mT±25mT while for the blue trace we have H_{res} = 340mT±5mT and ΔH = 154mT±25mT.

The resonance appears as a peak in the blue trace and a dip in the black trace. This can in fact be understood by looking at Supplementary Figure 4b: As explicated in the main text, at the resonance, some of the microwave radiation is absorbed by the quasiparticle spins and so less is transmitted to the detectors. We can see from Supplementary Figure 4b that at V_{DC}= -288μV a smaller effective V_{RF} gives a higher conductance, whereas at V_{DC}= -100μV a smaller effective V_{RF} gives a lower conductance, hence the different in the sign of the resonance in the two traces. To optimise sensitivity for this detection scheme, V_{DC} and V_{RF} should be chosen so that $\mathrm{d}G/\mathrm{d}V_{\mathrm{RF}}$ is maximal.

Supplementary Note 4: Estimate of the Number of Quasiparticles

The switching current I_{s} of the Al bar in the absence of microwaves and of quasiparticle injection is ~1800μA (Figure 1f of the main text): we remind the reader that, for the blue trace, current is injected along the length of the S bar. Detection Scheme 1 should be close to this 'equilibrium' situation as the voltages applied across the NIS junctions are of the order of the superconducting gap Δ (at zero temperature).

In contrast, in Detection Scheme 2 (Figure 1f and Figure 3 of the main text), current is injected into the S bar across a tunnel junction and 'removed' via another such junction, e.g. as shown in Figure 1d of the main text. Here, the voltages across the NIS junctions (which typically

have resistances of ~5kΩ) at the point where the S bar becomes normal are several mV and we expect the quasiparticles in the S bar to be driven strongly out-of-equilibrium by the injected current. Typical I_s measured are around 500-600nA.

If we assume that the non-equilibrium quasiparticle population can be described by an effective temperature T_eff, and that I_s scales with T_eff in the same way that Δ does, then in DS2 $T_\mathrm{eff} > T_\mathrm{c}/2$, with T_c being the critical temperature. Based on Figure 4 of Ref. [6], we can then say that the quasiparticle density in DS2 is at least two orders of magnitude higher than in DS1.

Supplementary References

[1] Jedema, F. J., Heersche, H. B., Filip, A. T., Baselmans, J. J. A. & Wees, B. J. v. Electrical detection of spin precession in a metallic mesoscopic spin valve. *Nature* **416**, 713–716 (2002).

[2] Anthore, A., Pothier, H. & Esteve, D. Density of States in a Superconductor Carrying a Supercurrent. *Physical Review Letters* **90**, 127001 (2003).

[3] Tinkham, M. *Introduction to Superconductivity* (Dover, Mineola, 1996), 2 edn.

[4] Fulde, P. High field superconductivity in thin films. *Advances in Physics* **22**, 667–719 (1973).

[5] Quay, C. H. L., Dutreix, C., Chevallier, D., Bena, C. & Aprili, M. Frequency-domain measurement of the spin imbalance lifetime in superconductors. *arXiv:1408.1832 [cond-mat]* (2014).

[6] de Visser, P. J. *et al.* Number fluctuations of sparse quasiparticles in a superconductor. *Physical Review Letters* **106**, 167004 (2011).
